# Clinical, lifestyle, socioeconomic determinants and rate of asymptomatic intracranial atherosclerosis in stroke free Pakistanis

**DOI:** 10.1186/s12883-014-0155-6

**Published:** 2014-08-15

**Authors:** Ayeesha Kamran Kamal, Farzin Majeed, Omrana Pasha, Hasan Rehman, Muhammad Islam, Iqbal Azam, Muhammad Saleem Ilyas, Munawar Hussain, Kamran Masood, Bilal Ahmed, Sumaira Nazir, Zafar Sajjad, Scott E Kasner

**Affiliations:** 1 Neurology, Stroke Service, The International Cerebrovascular Translational Clinical Research Training Program (Fogarty International Center, National Institutes of Health) and Aga Khan University, Karachi, Pakistan; 2 Fogarty Cerebrovascular Research Fellow, The International Cerebrovascular Translational Clinical Research Training Program (Fogarty International Center, National Institutes of Health) and Aga Khan University, Karachi, Pakistan; 3 Associate Professor, Director Masters in Epidemiology and Biostatistics Program, Community Health Sciences, Aga Khan University, Karachi, Pakistan; 4 The International Cerebrovascular Translational Clinical Research Training Program (Fogarty International Center, National Institutes of Health) and Aga Khan University, Karachi, Pakistan; 5 Department of Community Health Sciences, Aga Khan University, Karachi, Pakistan; 6 Dow University of Health Sciences, Karachi, Pakistan; 7 Dow Institute of Radiology, Dow University of Health Sciences, Karachi, Pakistan; 8 Epidemiology and Biostatistics, Department of Medicine, Aga Khan University, Karachi, Pakistan; 9 Radiology, Aga Khan University, Karachi, Pakistan; 10 Division of Stroke and Neurointensive Care, Comprehensive Stroke Center University of Pennsylvania, Philadelphia, Pennsylvania, USA

**Keywords:** Intracranial atherosclerosis, Stroke, Asymptomatic, Developing countries, Prevention, Sociodemographic risk factors, Epidemiology

## Abstract

**Background:**

Intracranial Atherosclerotic Disease (ICAD) is the most frequent etiology of stroke with high prevalence among Asians. Despite this, early determinants of ICAD have not been described from this region.

**Methods:**

The study is an analytical prospective cross-sectional study of 200 adults from Radiology Departments of two diagnostic centers in Karachi. Eligible participants confirmed the absence of stroke symptoms via the Questionnaire for Verifying Stroke Free Status (QVSFS) and underwent an interview covering medical, socio demographic, lifestyle and anthropometric evaluation using locally validated and standardized definitions. Magnetic Resonance Images (MRI) were centrally reviewed to detect ICAD using the criterion used in the Warfarin–Aspirin Symptomatic Intracranial Disease study. The risk factors associated with asymptomatic ICAD are reported along with prevalence ratios.

**Results:**

Of the 200 participants, ICAD was found in 34.5% (n = 69) of the participants. Mean age was 37.1 (S.D 15.1) years with 62% younger than 45 years. Self-reported hypertension was found in 26.5% subjects, diabetes in 9%, dyslipidemia in 5% and depression in 60%. Smokeless tobacco (Adjusted PR 3.27 (1.07-6.05)), Western diet, high socioeconomic status (Adjusted PR 2.26 (1.99-5.62)) and dyslipidemia (Adjusted PR 1.88 (1.25-2.21)) had significant associations with ICAD after multivariable analysis. Age, gender, diabetes, hypertension, depression and physical activity did not have a significant association.

**Conclusion:**

ICAD was found on MRI in one in three asymptomatic Pakistanis and was associated with modifiable risks. Initiatives targeting primary prevention may be able to decrease the burden of disease caused by stroke due to ICAD.

**Study Registration Number:**

NCT02072876 2/25/2014

## Background

Stroke is a leading cause of adult disability worldwide. It is also thought to be the 2nd most common cause of global mortality [1]. Among ischemic stroke sub-types, intracranial atherosclerotic disease (ICAD) is regarded as the most frequent etiology of stroke with high prevalence among Asian, African and Hispanic individuals [2]–[8]. Early data from Pakistan has also indicated a high prevalence of ICAD [9].

Symptomatic atherosclerotic disease is preceded by asymptomatic disease [10]. This provides us with an opportunity for prevention [11]. For ICAD, prevention is key since few relevant interventions exist once the disease is symptomatic, none of which are easily implementable in resource strapped regions [12]. However, in order to formulate effective mechanisms of preventing stroke, it is imperative to first identify the early determinants of ICAD. Although these determinants have been extensively studied in the Western population, data is not always applicable to a population such as the one in Pakistan due to different demographic and lifestyle characteristics. Despite the high prevalence of ICAD, data from Asian regions that bear large burdens of ICAD is relatively scarce.

The objective of this study was to determine the clinical, lifestyle, dietary and psycho-social determinants of asymptomatic ICAD in Pakistanis who do not report stroke symptoms on systematic review. These determinants would help identify high risk behavior for stroke in the Pakistani population.

## Methods

### Study design

This is a prospective cross-sectional study which was carried out between March and June 2013 at the Radiology Departments of Aga Khan University (AKU) and Dow University of Health Sciences (DUHS).

### Participating centers

Aga Khan University is a private not for profit academic center with a fee for service system while Dow University Radiology Center is public sector, government funded and subsidized entity. Radiology departments in both centers are equipped with 1.5 Tesla MRI scanners. The two centers are located in Karachi, Pakistan’s largest city with inhabitants of all ethnicities [13]. They are the two largest MRI scanning facilities in Karachi and perform greater than a thousand neurologic scans per month.

### Study population

We recruited adult Pakistani patients > 18 years of age who presented to the participating centers for MRI of the Brain for indications other than transient ischemic attack (TIA) or stroke (e.g. headaches, epilepsy, rhino sinusitis etc.). Subjects were required to have no history of stroke or TIA, confirmed by a negative result on the Questionnaire to Verify Stroke-free Status (QVSFS).

### Data collection procedures/study flow

Non-probability purposive sampling was used to recruit consecutive patients prospectively from the two sites. Participants were screened for the absence of stroke via QVSFS by trained medical officers after which an informed consent was taken for enrollment into the study [14],[15]. An already tested Urdu version of QVSFS was used [16]–[18]. Those who were QVSFS negative were subject to detailed interview and an additional 5 minute MRA without contrast was done. Images were then collected on Compact Disks (CD) by the designated research officers which were then reviewed centrally by the vascular team offline who were unaware of the patient’s clinical history. The team evaluating the images had extensive experience in systematically reviewing vasculature.

### Data variables

A data collection form (DCF) was used that identified and collected baseline demographic information. It also consisted of an instrument that was used to record findings from the MRI-MRA images. The instrument was adapted from the work of Wardlaw [19],[20] and it has been used in a study previously [9].

#### Demographic and socio-economic

We collected information about age, sex, ethnicity, education and marital status in addition to 5 questions regarding income, occupation, number of household members and household assets. For assessing household assets, the Assets Questionnaire developed by the World Bank for developing countries was used [21].

#### Principal component analysis (PCA) for socio-economic status

To classify economic status, the following socioeconomic variables were considered: individual’s profession, education, household income, number of people sharing a room in their house and household assets including property (19 items). A principal component analysis (PCA) was applied to these socioeconomic indicator variables, which showed relevant contributions (>10% and <70%) to the combined socioeconomic status score factor [22]. The factor of the PCA with the highest eigenvalue was used as the variable to describe sufficiently the socioeconomic status of a household. The respective factor scores were categorized into tertiles and used in the regression analysis. The lowest 33% of households according to the economic status variable were classified as belonging to low socioeconomic status, the highest 33% as high socioeconomic status and the rest as middle socio economic status [23].

#### Clinical

We inquired about clinical risk factors including diabetes, hypertension, dyslipidemia, ischemic heart disease and atrial fibrillation. Hypertension was defined as those with elevated systolic blood pressure >140 mmHg or diastolic blood pressure >90 mmHg [24], or those with a clear hypertension medical record, or record of antihypertensive drugs usage. Subjects with diabetes mellitus were those with documented diabetes in their medical records, those having a random glucose level of >11.1 mmol/L (200 mg/dl) or those on anti-diabetics [25]. Dyslipidemia was defined as total cholesterol >200 mg/dl or LDL (Low density lipo-protein) >160 mg/dl or those taking lipid-lowering drugs [26]. IHD (Ischemic heart disease) was defined as a known history of myocardial infarction or angina. Anthropometric indices were done for the measurement of general and central obesity. For categorizing BMI (Body mass Index) World Health Organization’s (WHO) recommendations for Asian population were used [27]. SRQ was used for assessing depression, its validity has been tested in Pakistani population and found to have good psychometric properties with sensitivity of 78% and specificity of 81% [17].

#### Assessment of dietary pattern

For the purpose of this analysis we divided dietary patterns as “prudent” and “western” diet based on the nature of the food stuff being consumed. These are descriptive labels for the purposes of this study only and do not generalize or imply dietary culture per se. Data was collected on a Food Frequency Questionnaire (FFQ) (Additional file 1).

Prudent diet pattern for this study represented high intakes of vegetables, fruit, legumes, fish, poultry, and whole grains, whereas Western diet for the purpose of analysis reflects high intakes of red meat, processed meat, refined grains, french fries, and sweets/desserts.

To identify dietary patterns, the items of the FFQs were first grouped into 29 food groups [28],[29]. The classification of food groups was based on similarities in nutrient profile and culinary preference. Factor analysis (principal component analysis) was then applied with the orthogonal rotation procedure varimax to the predefined food groups.

We determined the dietary patterns to retain based on the Scree test [30] (a graphical presentation of eigenvalues, with eigenvalues >1 explaining more variance than an individual food group) and the interpretability of factors. The Scree test allowed us to identify 2 major patterns with the largest eigenvalues (≥3.09). These patterns were labeled the “prudent” and the “Western” patterns as above. The scores of each of these patterns were then divided into quintiles with higher quintile representing higher adherence to that dietary pattern.

#### Assessment of physical activity

MET minutes/week score for walking, moderate and vigorous activity were calculated from IPAQ short by multiplying the total minutes per week of that activity by its designated MET score [31]. Total MET minutes per week were calculated by summing up the walking, moderate and vigorous MET minutes per week. This was then used to categorize physical activity into low, moderate and high according to the following criteria: <600 MET-minutes/ week as low physical activity, between 600 MET-minutes/week and 3000 MET-minutes/week as moderate physical activity and >3000 MET-minutes/week as high physical activity (Additional file 2).

#### Radiologic assessment

Intracranial Stenosis on MRA was defined as any artery (bilateral anterior cerebral arteries (A1 & A2), bilateral middle cerebral arteries (M1 & M2), bilateral posterior cerebral arteries, bilateral vertebral arteries, bilateral internal carotid artery- petrous bilateral internal carotid artery- cavernous, bilateral internal carotid artery- supraclinoid and basilar artery) showing abnormality when imaged by time of flight MRA method. Any degree of stenosis was classified as diseased. This ranged from atherosclerotic irregularity to complete occlusion. We used this stratification since data suggests that even minor degrees of ICAD can result in fatal strokes, mediated by platelet destabilization, plaque rupture etc. in addition to simple progressive atherosclerotic stenosis. Since which minimal stenosis can be potentially associated with clinically significant outcomes [32] we considered all biologically involved vessels.

An internationally standardized equation was used to calculate the degree of stenosis. This method has also been used successfully in a Pakistani study [9] to measure symptomatic intracranial stenosis.

(1) $$\mathrm{Percent}\;\mathrm{stenosis}\;=\;\left[1-\;\left(D\;\mathrm{stenosis}/D\;\mathrm{normal}\right)*100\right]$$

where D Stenosis is the diameter of the artery at the site of the most severe stenosis, and D normal is the diameter of the proximal normal artery [19].

We stratified the degree of stenosis to better understand the burden and distribution of ICAD in our population; we were not able to stratify our *analysis* according the degree of stenosis due to a small number of diseased arteries expected from our study.

### Statistical analysis

#### Sample size estimation

A minimum sample size of 200 participants was required in order to achieve 80% power for detecting a minimum difference of 20% in the prevalence of radiological findings between asymptomatic ICAD positive and ICAD negative persons. This was calculated assuming a 1:3 ratio in patients with ICAD versus no ICAD based on previous studies from Asia [33]–[36] and at a level of significance of 5%.

### Statistical analysis

Mean and standard deviations (SD) were calculated for continuous variables like age and BMI and proportions for categorical variables like education, ICAD, clinical and lifestyle factors. The independent contribution of any risk factor to ICAD was examined in the univariate and subsequent multivariable Cox proportional hazards algorithm and prevalence ratios and 95% confidence intervals were reported.

### Ethical approval and human subjects protection

All participants provided written informed consent. Ethical approval was taken from AKU Ethical Review Committee and the Institutional review board of DUHS. (ERC number 2327 CHS ERC 12 and IRB 360/DUHS 2012). All scans were reviewed within 24 hours by the radiology faculty and there was provision in the study to report any critical, life threatening incidental findings like aneurysms first to the referring physician and in his absence or inability to contact, the report was communicated to the patient with an urgent referral. All data was numerically coded so as not to reveal the identity of the participants. All electronic transfer within centers was done through decoded CDs that were centralized to a single password protected workstation for analysis.

## Results

**Figure 1:**
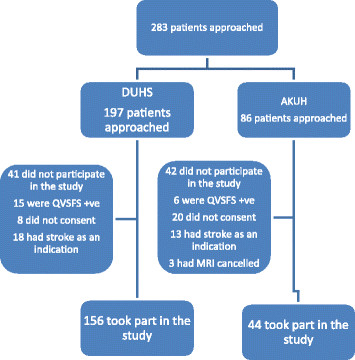
Study flow diagram.

Out of 283 subjects approached at the two study sites (197 at DUHS, 86 at AKUH), 200 were recruited for this study (156 at DUHS and 44 at AKUH). Of those excluded from the study most patients had stroke as an indication for MRI (37.3%) or did not consent for the study (33.7%) (Figure 1). The most common indication for the MRI was headache (52%). Out of the 200 who participated in the study, 69 (34.5%) were found to have clinically significant ICAD.

### Demographics

Sociodemographic details of study participants are presented in Table 1. Mean age of the participants was 37.1 years (S.D 15.1) years with nearly 62% of the participants being younger than 40 years. There was an equal proportion of males and females. The largest ethnic group was the Urdu speaking community (37%, n = 74) followed the Punjabi community. There were no significant differences between the age, gender or ethnic distribution between patients with and without ICAD. Although the proportion of low socioeconomic status was roughly equal in the two groups (34.8%, n = 24 and 32.1%, n = 42), the proportion of high socioeconomic status of the subjects with ICAD was higher in patients with ICAD (42%, n = 29) compared to those without ICAD (29%, n = 38). Most participants understandably belonged to Karachi with relatively small proportions of participants from other cities. Subjects with ICAD tended to be more educated than the ones without ICAD (37.7% with ICAD were graduates compared to 24.4% without ICAD).

**Table 1:** Socioeconomic and demographic profile of study participants

**SOCIO-ECONOMIC FACTORS**	**Frequency in patients with ICAD 69 (34.5%) n (%)**	**Frequency in patients with no ICAD 131 (65.5%) n (%)**
**Age (Years)**		
Mean (SD)	38.6 (15.1)	36.31 (15)
**Gender**		
Males	36(52.2%)	65(49.6%)
Females	33 (47.8%)	66 (50.4%)
**Ethnicity**		
Urdu speaking	28 (40.7%)	46 (35%)
Pathan	17 (24.6%)	26 (19.8%)
Sindhi	6 (8.7%)	23 (17.6%)
Balochi	4 (5.8%)	11 (8.4%)
Punjabi	7 (10.1%)	8 (6.1%)
Others	7 (10.1%)	17 (13%)
**Education**		
Illiterate	16 (23.2%)	30(22.9%)
Madressa	6 (8.7%)	13 (10%)
Primary	9 (13%)	11 (8.4%)
Secondary	12 (17.4%)	45 (34.4%)
Graduate and above	26(37.7%)	32 (24.4%)
**Occupation**		
Employed	26 (37.7%)	30 (23%)
Unemployed	3 (4.3%)	12 (9.2%)
Retired	2 (2.9%)	4 (3.1%)
Housewife	23 (33.3%)	60 (45.8%)
Laborer	5 (7.2%)	9 (6.9%)
Business and others	10 (14.5%)	16 (12.2%)
**Socio-economic status**		
Low	24 (34.8%)	42 (32.1%)
Middle	16 (23.2%)	51 (38.9%)
High	29 (42%)	38 (29%)
**Indications for MRI**		
Headache	34 (49.3%)	71 (54.2%)
Dizziness	0	8 (6.1%)
Headache & dizziness	1 (1.4%)	9 (7%)
Tremors	1 (1.4%)	4 (3.1%)
Seizures	3 (4.3%)	4 (3.1%)
Others	32 (46.5%)	35 (26.5%)
**City of residence**		
Karachi	25 (36.2%)	51 (39%)
Quetta	9 (13%)	20 (15.2%)
Hyderabad	10 (14.5%)	15 (11.4%)
Gilgit	8(11.5%)	9 (6.8%)
Other cities	17 (24.6%)	36 (27.4%)

### Risk factors

Table 2 shows the risk factors of stroke in study participants with and without ICAD. Among the clinical determinants of ICAD, hypertension (26.5% of subjects, n = 53) was most commonly found followed by diabetes (9%, n = 18) There were no major differences in the proportions of subjects with and without ICAD in any of the diseases mentioned above. Dyslipidemia however was significantly associated with ICAD (p value: 0.04).CVD and Atrial fibrillation were present in very low proportions in both groups and did not show any significant associations with ICAD.

**Table 2:** Risk factors for asymptomatic ICAD

**RISK FACTORS**	**Frequency of patients with ICAD (%)**	**Frequency of patients without ICAD (%)**	**Unadjusted PR (CIs)**	**Adjusted PR (CIs)**	**P-value (<0.05 sig.)**
**Socioeconomic status**					
Low	24 (34.8%)	42 (32.1%)	1	1	0.05
Middle	16 (23.2%)	51 (38.9%)	0.66 (0.35-1.24)	0.67 (0.33-1.35)	.07
High	29 (42%)	38 (29%)	1.21 (0.70-2.08)	2.26 (1.99-5.62)	0.03
**Clinical risk factors**					
Diabetes	4 (5.8%)	14 (10.7%)	0.62 (0.23-1.70)	0.58 (0.2-1.67)	0.27
Hypertension	17 (24.6%)	36 (27.5%)	0.97 (0.84-1.12)	0.98 (0.89-1.08)	0.76
Dyslipidemia	7 (10.3%)	3 (2.2%)	1.16 (0.37-3.70)	1.88 (1.25-2.21)	0.04
CVD	2 (2.9%)	1 (.8%)	0.51 (0.13-2.09)	0.30 (0.06-1.43)	0.13
A.Fib	1 (1.4%)	2 (1.5%)	1.04 (0.15-7.49)	1.44 (0.17-3.39)	0.73
**Lifestyle factors**					
Current smoking	5 (7.2%)	11 (8.4%)	1.12 (0.45-2.78)	1.73 (0.24-2.27)	0.59
Past smoking	3 (4.3%)	10 (7.6%)	1.54 (0.48-4.89)	2.19 (0.51-3.81)	0.30
Smokeless tobacco	19 (27.9%)	5 (3.8%)	1.76 (0.71-4.36)	3.27 (1.07-6.05)	0.02
**BMI categories**					
Normal	11 (16%)	15 (11.5%)	1	1	0.67
Underweight	20 (29%)	39 (29.8%)	1.30 (0.62-2.71)	1.73 (0.77-3.88)	0.21
Pre-over weight	10 (14.5%)	20 (15.3%)	0.98 (0.46-2.10)	0.92 (0.45-1.84)	0.64
Over-weight	14 (20.3%)	34 (26%)	0.86 (0.44-1.70)	1.18 (0.54-2.60)	0.75
Obese	13 (18.8%)	23 (17.6%)	1.07 (0.53-2.14)	1.13 (0.53-2.38)	0.70
**Central obesity**					
Waist	19 (27.5%)	30 (23%)	1.20 (0.70-2.03)	1.14 (0.64-2.03)	0.65
Circumference > 80 cms					
**Physical activity**					
Low	56 (81.2%)	106 (81%)	1	1	0.84
Moderate	3 (4.3%)	6 (4.6%)	0.96 (0.30-3.06)	1.04 (0.32-3.41)	0.09
High	10 (14.5%)	19 (14.5%)	0.99 (0.51-1.94)	1.25 (0.59-2.65)	0.54
**Western diet**					
Quintile 1	6 (8.7%)	31 (23.7%)	1	1	0.15
Quintile 2	16 (23.2%)	23 (17.6%)	2.46 (0.96-6.29)	2.24 (0.79-6.39)	0.10
Quintile 3	18 (26%)	21 (16%)	2.77 (1.10-6.98)	2.58 (0.95-2.61)	0.14
Quintile 4	13 (18.8%)	26 (19.8%)	2.01 (0.76-5.26)	2.86 (1.11-7.41)	0.02
Quintile 5	13 (18.8%)	25 (19%)	2.05 (0.78-5.40)	3.04 (1.15-7.99)	0.03
**Prudent diet**					
Quintile 1	11 (16%)	26 (19.8%)	1	1	0.47
Quintile 2	13 (21.5%)	28 (21.4%)	0.95 (0.41-2.19)	1.02 (0.42-2.4)	0.95
Quintile 3	7 (12%)	30 (22.9%)	0.78 (0.32-1.87)	0.76 (0.31-1.92)	0.57
Quintile 4	9 (13%)	9 (7%)	1.68 (0.69-4.06)	1.39 (0.55-3.49)	0.48
Quintile 5	25 (36.2%)	33 (25.2%)	1.48 (0.73-2.99)	1.5 (0.71-3.18)	0.29
**Depression**	39 (56.5%)	81 (61.8%)	0 .85 (0.53-1.37)	0.91 (0.54-1.53)	0.17

Similarly, the physical activity level in the two groups was almost identical with over 80% of participants in both groups having low physical activity levels. Mean BMI was 25 kg/m^2^ (S.D 6.4 kg/m^2^) and mean waist circumference was 77 cm (S.D 14.88 cm). Using the cutoffs recommended for South Asians [25], 42% (n = 84) of the participants were above normal BMI range (obese + overweight). There were however no significant differences between the two groups.

The diet of the study participants varied between the groups with and without ICAD. Patients with ICAD were more likely to be on a higher quintile of Western and prudent diet as compared to those without ICAD.

Among the lifestyle determinants of ICAD, 12% of the study participants had a history of smokeless tobacco use (mostly niswar) with only 8% being current smokers. Understandably, the subjects with ICAD had a significantly higher proportion of using smokeless tobacco compared to those without ICAD (p value:0 02).

The frequency of depression (SRQ cut-off > 8) in the current sample of participants was 60%, even though a high cut-off point was used rather than the mean score. The proportion of subjects with (56.5%, n = 39) and without ICAD (61.8%, n = 81) however had no significant differences (p value: 0.17).

When multivariable cox regression analysis was applied, smokeless tobacco (PR 3.27; 95% CI 1.07-6.05) and higher quintiles of western diet (PR 3.04; 95% CI 1.15-7.99) were found to be significantly associated with ICAD. All biologically plausible interactions between clinical and lifestyle factors were checked but no significant interactions were found.

After further adjustment for the clustering effect (study centers) younger age (<45 years) was found to be significantly associated with ICAD (PR 1.94; 95% CI 1.85-1.97). Among the vascular risk factors presence of cardiovascular disease (PR 1.15; 95% CI (1.06-1.36) came out to be significantly associated with asymptomatic disease, besides lifestyle factors of increased consumption of western diet and smokeless tobacco use (Table 3).

**Table 3:** Multivariable model adjusted for clustering effect (Study Centers)

**Factor**	**Unadjusted PR**	**Adjusted PR**	**P-value**
**Sociodemographic factors**			
Age < 45 yrs	1.16 (1.13-1.19)	1.94 (1.85-1.97)	0.03
**Socioeconomic Status**			
Low	1	1	0.01
Middle	0.65 (0.34-1.24)	0.55 (0.51-0.59)	0.36
High	1.16 (1.08-1.41)	1.39 (1.08-1.83)	0.02
**Vascular related**			
HTN	1.96 (1.83-2.13)	-	
CVD	1.51 (1.39-1.64)	1.15 (1.06-1.36)	0.03
**Lifestyle related**			
Smokeless tobacco	1.73 (1.57-1.89)	1.77 (1.54-2.03)	0.04
Waist	1.22 (1.20-2.24)	-	
**Western diet**			
Quintile 1	1	1	0.01
Quintile 2	2.4 (2.2-2.6)	2.85 (2.06-3.95)	0.03
Quintile 3	2.46 (2.44-2.48)	2.87 (1.79-4.6)	0.03
Quintile 4	2.95 (1.78-4.87)	3.54 (2.68-4.68)	0.02
Quintile 5	3.32 (2.8-3.9)	3.9 (2.87-5.3)	0.01

## Discussion

To the best of our knowledge this is the first study conducted in South Asia that identifies early determinants of ICAD by looking at asymptomatic subjects. The major finding of this study is that modifiable risk factors such as dyslipidemia, tobacco chewing and overconsumption of a Western diet, which are all increasing in prevalence in our population are associated with asymptomatic ICAD. The most commonly affected arteries were the Posterior Cerebral arteries followed by Vertebral and Middle Cerebral arteries. Of all affected arteries, about 20% showed complete occlusion in clinically silent areas. Furthermore, it also showed a high prevalence of depression and sedentary lifestyle in the study population, both of which are stroke risk factors. The findings provide us with direction for implementation of initiatives that target primary prevention.

The proportion of patients with ICAD (34.5%) was significantly higher than that reported in previous studies from other parts of the world [37]–[39] that have reported figures between 4% and 29%. Although a figure as high as this cannot be ignored, the recruitment bias introduced choosing participants with a preexisting indication for an MRI would suggest that this figure might be falsely elevated.

The study had a young (mean age 37.1 yrs) population in general but importantly there was no significant difference in the ages of the subjects with and without ICAD. The finding is not consistent with previous similar studies [10],[35],[40],[41] from other parts of the world which report an association between increasing age and ICAD. However, this observation can still be explained if the findings of a previous study on the Pakistani population [16] is taken into consideration. Atherosclerotic disease takes years to develop and the study mentioned above showed the mean age of patients of symptomatic ICAD to be 50.1 years. This essentially suggests that a significant number of young apparently healthy Pakistanis have asymptomatic ICAD which can eventually progress to symptomatic ICAD. Further, ICAD is not entirely explained by traditional risks like hypertension, diabetes, and dyslipidemia, and emerging factors like plasma homocysteine, angiotensin-converting enzyme polymorphism, and inflammatory cytokines may affect its development. These biochemical investigations were beyond the capacity of this study [42]–[44].

Stroke is common in males and this observation is more pronounced in ischemic strokes [45]–[47]. It also occurs at a younger age in males compared to females [48],[49]. With a young study population, as in this study, the same finding would be expected. This study however does not report a gender predilection. This finding, although not common, has been shown in previous studies [35],[40] from other Asian countries. Interestingly a study [50] by Jafar et al. on 3143 Pakistani adults did report that women in Pakistan were at higher risk than men for coronary artery disease due to higher number of risk factors (diabetes, hypertension, obesity, dyslipidemia). Since the same risk factors as applicable to ICAD, we theorize that the equal risk of vascular disease observed in the cardiac population also applies to the cerebrovascular risk patients.

When looking at the lifestyle factors, our study findings suggest that a high socioeconomic status is significantly associated with stroke. Review studies [51],[52] that have specifically addressed the association between socioeconomic status and stroke have documented that it is in fact low socioeconomic status that is associated with higher incidence and severity of stroke. We suggest that lifestyle choices associated with the higher socioeconomic status such as adoption of Western diet have contributed to a higher incidence of stroke in our transitional population [53]. What adds weight to this argument is that the western diet has also been shown to be significantly associated with ICAD in our study (Adjusted PR 2.86 (1.11-7.41) for quintile 4 and Adjusted PR 3.04 (1.15-7.99) for quintile 5). There is documented evidence of the proinflammatory effects of Western diet [54]–[57] especially when taken in large quantities. The finding emphasizes the need to address the adaptation of Western diet before it adds to the burden of non communicable diseases in Pakistan which is now becoming endemic.

The use of smokeless tobacco is Pakistan is rampant with an estimated prevalence between 16% and 26% [58]–[61]. Overall prevalence of smokeless tobacco use was 12% in our study which was higher in participants with ICAD compared to those without. The association was of ICAD was found to be significant after multivariate analysis (Adjusted PR 3.27) reiterating the increased risk of stroke with use of smokeless tobacco which has documented before [62].

The causal relationship of diseases such as dyslipidemia, hypertension and diabetes with ICAD is well studied and hence we did not look to screen patients for these diseases. The presence or absence of these diseases was mostly based on history and a single blood pressure reading and random blood sugar test. The ascertainment bias resulting from this methodology could have contributed to the finding that apart from dyslipidemia, no other diseases such as hypertension and diabetes showed an association with ICAD. At the same time what also needs to be considered is the fact that a lot of these subjects were young and they might develop these diseases in the future and ICAD has simply manifested earlier due to lifestyle choices.

The association of depression with intracranial disease has been studied before and it has been suggested that small brain infarctions can cause depression [63],[64]. Through our study we attempted to establish an association, if any, of ICAD and depression. Recent data [65]–[68] reports the prevalence of depression among the residents of Karachi between 16% and 70%. Taking this into consideration we used a high cut off score for the SRQ (>8) to increase the specificity (Positive predictive value is 98% at this SRQ score). The study however still showed depression in more than half the subjects. With such a high figure, distributed equally across the study population, it is very difficult to establish any association. Similarly, more than 80% of the participants had a low physical activity level. With a prevalence as high as this, it is not possible to establish an association.

We feel that our preliminary observations have impact and relevance in a global context.

In a resource strapped country like Pakistan, we feel the key to tackling a health issue like ICAD lies in primary prevention. Evidence currently available for the therapies of ICAD [69]–[73] from developed countries shows that these interventions are not only expensive but often ineffective, and difficult to execute at best in low middle income settings.

Our suggestion to focus on controlling non communicable risk factors to reduce the burden of disease is not novel to low middle income regions. Our review of these initiatives reveals interventional projects in similar settings that addressed non communicable diseases and showed promising results [74]–[79]. What is lacking are holistic, stroke based or multiple risk initiatives.

Our study has several strengths. Our participants were chosen from the general population rather than from a high risk population. A detailed review of asymptomatic status was done by using a validated questionnaire (QVSFS) which to rule out clinical stroke with 95% sensitivity and specificity. This tool has also been used in Pakistan previously [16]. Rigorous training was given to all data collectors so that a standard protocol was implemented at both centers. A round the clock back up was available by stroke neurologists further adding clarity to the participant stroke free status. Both study centers were also equipped with a similar MRI scanner (1.5 Tesla) using standardized protocol thus reducing detection bias. Centralized coding system for MRA and specialized software was used for storing and image viewing. This study also assessed a number of novel risk factors for ICAD like socio-demographic factors, physical activity, diet and depression that have not always been explored using previously validated tools.

Our study has certain limitations. Since this is a cross sectional study, temporality cannot be assessed and no conclusions can be deduced regarding causality.There is a recruitment bias when we selected our participants from those who had a preexisting indication for an MRI. Secondly, the medical risks reported here (Hypertension and Diabetes) are self-reported, so that there may have been under-reporting and recall bias. This study also has a diagnostic limitation since we did not use a 4 vessel angiogram for diagnosing ICAD. This is because 4 Vessel angiography has a 1.5 - 3% risk of stroke [80]. Using this on otherwise healthy stroke free patients would be unethical. We used the MRI instead, which is a noninvasive tool with a high negative predictive value [81]. Using biochemical investigations such as homocysteine levels would have been relevant to identifying determinants of ICAD; however this was beyond the scope of this study, as was the opportunity to do risk loading stratified analysis based on degree of stenosis or the presence or absence of stroke and stroke brain volumes, both of which are avenues for further investigations. Furthermore, our data essentially applies to urban transitional Pakistan since rural based populations are not well represented in this study.

## Conclusion

In conclusion, we have demonstrated that the distribution of ICAD in young Pakistanis is frequent and equally distributed across gender and is associated with modifiable risks. Future studies should focus on longitudinal assessment of ICAD, and holistic risk reduction based interventions on younger populations.

## Abbreviations

ICAD: Intracranial Atherosclerotic disease

AKU: Aga Khan University

DUHS: Dow University of Health Sciences

MRI: Magnetic Resonance Imaging

TIA: Transient ischemic attack

QVSFS: Questionnaire to Verify Stroke-free Status

CD: Compact Disks

DCF: Data collection form

LDL: Low density lipo-protein

IHD: Ischemic heart disease

BMI: Body mass Index

WHO: World Health Organization’s

SRQ: Self-reported questionnaire

FFQ: Food Frequency Questionnaire

MET: Metabolic Equivalent

IPAQ: International Physical Activity Questionnaire

MRA: Magnetic Resonance Angiography

SD: Standard deviation

ERC: Ethical Review Committee

PR: Prevalence Ratio

## Competing interests

The authors declare that they have no competing interests.

## Authors’ contributions

AKK and FM jointly conceived the study, performed data analysis and wrote the first draft. OP advised on design issues, MI, IA, BA provided statistical input, MSI, MH, KM assisted and provided intellectual input for the study from the public sector perspective and radiologic collaborative input , SN provided analytical support, ZS provided radiologic support for center, SK provided international intellectual input, perspective and feedback. All authors read and approved the final manuscript.

## Authors’ information

Ayeesha Kamran Kamal and Farzin Majeed is Joint First Authors.

## Additional files

## Supplementary Material

Additional file 1: Dietary Assessment Through Food Frequency Questionnaire (FFQ). Click here for file

Additional file 2: IPAQ Calculation Algorithm. Click here for file
