# Supplementary material for: Clinical, lifestyle, socioeconomic determinants and rate of asymptomatic intracranial atherosclerosis in stroke free Pakistanis
Source: BMC Neurol. 2014 Aug 15;14:155. doi: 10.1186/s12883-014-0155-6 (PMC4236665; doi:10.1186/s12883-014-0155-6)
Supplement: Additional file 1: — Dietary Assessment Through Food Frequency Questionnaire (FFQ). [file s12883-014-0155-6-S1.docx]

**Additional File 1**

**Dietary Assessment through Food Frequency questionnaire (FFQ)**

I am going to ask you what you normally eat. Please estimate the no. of times you have eaten the following foods on average in the last month..

**Section 1: Staples**

| **Staples** | **Medium Serving** | **How often?**  **No. of times per:**  **D=Day, W=week,**  **M=Month, N=Rarely / Never** | | | |
| --- | --- | --- | --- | --- | --- |
| **BREADS** |  | D | W | M | N |
| Chappati/roti with fat* | 1 chappati |  |  |  |  |
| Chappati /roti without fat | 1 chappati |  |  |  |  |
| White bread | 1 slice |  |  |  |  |
| Wholemeal bread | 1 slice |  |  |  |  |
| Naan | 1 naan |  |  |  |  |
| Paratha | 1 paratha |  |  |  |  |
| Puri | 1 puri |  |  |  |  |
| Bajra rotla | 1 rotla |  |  |  |  |
| **CEREALS** |  | D | W | M | N |
| Low fiber/sugared cereals e.g. cornflakes | 1 bowl |  |  |  |  |
| High fiber cereals e.g.weetabix | 1 bowl |  |  |  |  |
| Dhuri (porridge) / Dariya | 1 bowl |  |  |  |  |
| Sweetbreads e.g. pancakes, muffins | 1 sweetbread |  |  |  |  |
| **DAIRY PRODUCTS** |  |  |  |  |  |
| Butter/Makkhan/  Margarine | 1 tsp |  |  |  |  |
| Whole Milk | 1 glass |  |  |  |  |
| Skimmed milk | 1 glass |  |  |  |  |
| Cream | 1 tsp |  |  |  |  |
| Cheese | 1 slice/ cube |  |  |  |  |
| Eggs (as white or whole ) | 1 |  |  |  |  |
| Lassi | 1 glass |  |  |  |  |
| Curd | ½ cup |  |  |  |  |
| **RICE** |  | D | W | M | N |
| Rice-white boiled only | 1 serv |  |  |  |  |
| Rice-white cooked with oil | 1 serv |  |  |  |  |
| Vegetable Pulao/Tehri | 1 serv |  |  |  |  |
| Meat Pulao/ Ukni | 1 serv |  |  |  |  |
| Meat Biryani | 1 serv |  |  |  |  |
| Khicheri (rice& lentils) | 1 serv |  |  |  |  |
| Dossa | 1 serv |  |  |  |  |
| Noodles(Chinese) | 1 serv |  |  |  |  |

**Section 2: Lentils, pulses, daals**

| **LENTILS, PULSES,**  **DaaLS** | **Medium Serving** | **How often?**  **No. of times per:**  **D=Day, W=week,**  **M=Month, N=Rarely / Never** | | | |
| --- | --- | --- | --- | --- | --- |
|  |  | D | W | M | N |
| Masoor dhal (red lentil) | 1 serv |  |  |  |  |
| Channa dhal (split peas) | 1 serv |  |  |  |  |
| Mung dhal (green lentil) | 1 serv |  |  |  |  |
| Pakoray, other basen products | ! serv |  |  |  |  |

**Section 3: Meat and fish**

| **MEAT AND FISH** | **Medium Serving** | **How often?**  **No. of times per:**  **D=Day, W=week,**  **M=Month, N=Rarely / Never** | | | |
| --- | --- | --- | --- | --- | --- |
| **SNACKS** |  | **D** | **W** | **M** | **N** |
| Burger / McDonalds | 1 burger |  |  |  |  |
| (Kentucky) Fried Chicken | 1 serv |  |  |  |  |
| Kebab | 1 kebab |  |  |  |  |
| Meat Samosa | 1 samosa |  |  |  |  |
| Lasagne | 1 serv |  |  |  |  |
| Pizza | 1 piece |  |  |  |  |
| **ROAST/ GRILLED /**  **BBQ** |  | **D** | **W** | **M** | **N** |
| Chicken/ Tikka | 1 serv |  |  |  |  |
| Lamb / Chops | 1 serv |  |  |  |  |
| Beaf/ Steak | 1 serv |  |  |  |  |
| **CURRIES** |  |  |  |  |  |
| Chicken & potato or other veg | 1 serv |  |  |  |  |
| Chicken alone/Karai/Bhunna | 1 serv |  |  |  |  |
| Meat & potato/Aloo Ghost | 1 serv |  |  |  |  |
| Meat alone-lamb,mutton, beaf,chops/Karai/Bhunna | 1 serv |  |  |  |  |
| Ghost palak/meat & other veg | 1 serv |  |  |  |  |
| Kofta | 1 serv |  |  |  |  |
| Keema | 1 serv |  |  |  |  |
| Aloo Keema | 1 serv |  |  |  |  |
| Keema matar | 1 serv |  |  |  |  |
| **FISH DISHES** |  | **D** | **W** | **M** | **N** |
| Masala fried fish | 1 serv |  |  |  |  |
| Fried fish with batter, as in fish and chips | 1 serv |  |  |  |  |
| Fish fingers | 1 serv |  |  |  |  |
| Other white fish-fresh or frozen | 1 serv |  |  |  |  |
| Shell fish e.g. crab, prawns | 1 serv |  |  |  |  |

**Section 4: Fruits & Salads**

|  |  | **D** | **W** | **M** | **N** |
| --- | --- | --- | --- | --- | --- |
| Fresh Fruit/s | Y / N |  |  |  |  |
| **If yes, What kind of fruit? How much?**  *Write 1 if you ate one apple, h½ if you ate a half* | **Medium serving** | **D** | **W** | **M** | **N** |
| Apple | _____ piece |  |  |  |  |
| Banana | _____ piece |  |  |  |  |
| Orange | _____ piece |  |  |  |  |
| Melon | _____ slice |  |  |  |  |
| Fruit salad | _____ serv |  |  |  |  |
| Other fruit ( specify) | ____piece/portion |  |  |  |  |
| Fruit Juices | _____ glass |  |  |  |  |
| Vegetable/Green salad | ______ serv |  |  |  |  |
| Coleslaw | ______ serv |  |  |  |  |

**Section 5: Confectionaries/Bakery Items**

|  | **Medium serving** | **D** | **W** | **M** | **N** |
| --- | --- | --- | --- | --- | --- |
| Regular biscuits |  |  |  |  |  |
| Low sugar biscuits |  |  |  |  |  |
| Cake/Brownie/Pastry/Doughnuts | 1 slice |  |  |  |  |
| Chocolates | 1 bar |  |  |  |  |
| Ice-cream | 1 cup |  |  |  |  |
| Crisps/Chips | 1 serv |  |  |  |  |
| French fries | 1 serv |  |  |  |  |

**Description of food groups in the Food Frequency Questionnaire**

**a) Meat/poultry:** Includes beef, lamb, mutton, goat, chicken

**b) Fish:** Includes fresh-water and sea-water fish; canned fish, dried fish.

**c)** **Prawns**

**d) Eggs:**

**d) Grains:** Includes whole wheat flour; whole wheat chappati, paratha, puri, wheat, rice, corn flour/maize, oats

**e) Refined/milled grains:** Includes white flour; white flour chapati; white/polished rice; pasta; noodles

**f) Dairy products:** Includes milk, yogurt, cheese, curd, raita, lassi, custard, khoya, firni, kheer, milk puddings, and ice cream.

**g) Deep fried foods:** Includes french fries, potato chips, samosas, papad, pakoras kebabs.

**h) Desserts/sweet snacks:** Includes the use of jam; cakes; pies; chocolate; candy; burfi/ladoo; rasgulla/gulab jamun; halwa; shameia, mohalabeia, Coke and other soft drinks.

**i) Legumes:** Includes dried beans, lentils, peas, daals; **o) Nuts/seeds:** Includes peanuts, almonds, sunflower seeds, cashews, walnuts.

**j) Fruit/juice:** Includes all fruits and their juices.

**k) Leafy greens:** Includes all fresh leafy green vegetables: spinach,

**l) Other raw vegetables:** Includes any raw vegetables not included in the preceding categories.

**m) Other cooked vegetables:** Includes any cooked vegetables not included in the preceding

categories.

| **Prudent Diet** | **Western diet** |
| --- | --- |
| Whole meal bread | Roti with fat |
| Naan | Paratha |
| Roti without fat | Butter |
| White bread | Cream |
| Cornflakes | Cheese |
| Whole Milk | Fried rice |
| Eggs | Lazania |
| Curd | Pizza |
| Daal | Noodles |
| Chicken Tikka | Meat Karahi |
| Chicken Biryani | Meat Kofta |
| Meat vegetable Curry | Prawns |
| Palak Ghost | Beaf Steak |
| Fish curry | Lamb chops |
| Boiled rice | Sweets |
| Green salad | Chocolates |
| Melon | Ice cream |
| Fruit juices | French fires |
|  |  |
