# Supplementary material for: Clinical, lifestyle, socioeconomic determinants and rate of asymptomatic intracranial atherosclerosis in stroke free Pakistanis
Source: BMC Neurol. 2014 Aug 15;14:155. doi: 10.1186/s12883-014-0155-6 (PMC4236665; doi:10.1186/s12883-014-0155-6)
Supplement: Additional file 2: — IPAQ Calculation Algorithm. [file s12883-014-0155-6-S2.docx]

## Appendix 1 -Scoring protocol for IPAQ short and long

Continuous score

a) IPAQ short:-

First the total minutes of walking, moderate and vigorous intensity activity per week was calculated by multiplying the minutes of an activity per day by the no of days per week the activity was reported.

- Walking MET-minutes/week = 3.3 * walking minutes per day * no of days per week in which walking was reported.
- Moderate MET-minutes/week = 4.0* moderate intensity minutes per day * no of days per week in which moderate intensity activity was reported.
- Vigorous MET- minutes per week = 8.0* Vigorous intensity minutes per day * no of days per week in which vigorous intensity activity was reported.
- Total physical activity MET-minutes/week = Walking + Moderate + Vigorous MET minutes/week scores.

The MET scores of 3.3, 4.0 and 8.0 for walking, moderate and vigorous intensity activity

were assigned as per the IPAQ scoring protocol.

Categorical score

Three categories of physical activity were defined for the IPAQ short.

Category 1 - Low physical activity level:

Those individuals who did not meet criteria for Categories 2 or 3 were put in this category and considered to have a ¡¥low¡¦ physical activity level.

Category 2- Moderate physical activity level:

- At least 20 minutes of vigorous intensity activity per day for three or more days per week OR
- At least 30 minutes of moderate intensity activity per day for 5 or more days per week

OR

- Five or more days of any combination of walking, moderate-intensity or vigorous intensity activities achieving a minimum Total physical activity of at least 600 METminutes/week.

Category 3- High Physical activity level

- Vigorous-intensity activity on at least 3 days achieving a minimum Total physical activity of at least 1500 MET-minutes/week OR
- Five or more days of any combination of walking, moderate-intensity or vigorous intensity activities achieving a minimum Total physical activity of at least 3000 MET-minutes/week.
